# Proportion of fentanyl reports in illicit drug seizures and nonfatal overdose emergency department visits in the United States, 2021–2024

**DOI:** 10.1016/j.drugpo.2026.105382

**Published:** 2026-06-06

**Authors:** Mehrdad Khezri, Saba Rouhani, Zoe Lindenfeld, Kaley Joss, Madalyn Huang, Jemar R. Bather

**Affiliations:** a Department of Epidemiology, NYU School of Global Public Health, New York, NY, USA; b Edward J. Bloustein School of Planning and Public Policy, Rutgers University, New Brunswick, NJ, USA; c Department of Biostatistics, NYU School of Global Public Health, New York, NY, USA

**Keywords:** Fentanyl, Synthetic opioids, Illicit drug supply, Drug seizures, Nonfatal overdose, Emergency department visits

## Abstract

**Background:**

The increasing presence of fentanyl in the illicit drug supply has been associated with rising overdose mortality in the United States, but the extent to which it is associated with nonfatal overdose morbidity remains unknown. We examined the association between the proportion of fentanyl reports in illicit drug seizures and state-level rates of nonfatal overdose emergency department (ED) visits.

**Methods:**

We conducted a longitudinal ecological analysis of 40 US states from 2021 to 2024. Outcomes were annual state-level rates of nonfatal overdose ED visits per 10,000 ED visits, obtained from Drug Overdose Surveillance and Epidemiology Syndromic Surveillance System. The proportion of fentanyl reports among all illicit drug seizure reports was obtained from the National Forensic Laboratory Information System. Annual state-level sociodemographic covariates were obtained from the American Community Survey. Adjusted associations and 95% confidence intervals (CIs) were estimated via linear regression using generalized estimating equations.

**Results:**

After adjusting for covariates, a 10 percentage-point increase in fentanyl seizure proportion was significantly associated with higher nonfatal overdose ED visit rates: opioid-involved (2.18 increase, 95% CI: 0.89, 3.46), fentanyl-involved (0.73 increase, 95% CI: 0.27, 1.18), and cocaine-involved (0.08 increase, 95% CI: 0.01, 0.15). There was no evidence of statistically significant associations with heroin-, stimulant-, methamphetamine-, and benzodiazepine-involved overdose ED visit rates.

**Conclusions:**

Greater fentanyl penetration in illicit drug seizure reports was significantly associated with higher opioid-, fentanyl-, and cocaine-involved nonfatal overdose ED visit rates across states. These findings suggest that fentanyl saturation is not only a driver of overdose mortality but also contributes to nonfatal overdose burden, with important implications for health system demand and public health preparedness.

## Introduction

Drug overdose remains a leading cause of morbidity and mortality in the United States (US) (Spencer et al., 2022). In recent years, the overdose crisis has increasingly been driven by synthetic opioids, particularly illicitly manufactured fentanyl and its analogs (Ciccarone, 2019; Pardo, Caulkins et al., 2019). These potent substances have saturated illicit drug markets, increasing overdose deaths and other adverse outcomes (e.g., HIV, HCV) (Ciccarone, 2019; O’Donnell, 2017). Although fentanyl initially emerged within heroin markets in the northeastern US, it has spread across regions and increasingly appears in non-opioid drug supplies, including cocaine and other stimulants (Pardo, Caulkins et al., 2019; Park et al., 2021). As a result, changes in the composition of the illicit drug supply have become a key structural driver of overdose risk in the fentanyl era.

Most research examining the relationship between fentanyl and overdose has focused on mortality outcomes (Bruzelius et al., 2024; Cano et al., 2023, 2024; Dahlen et al., 2026). However, nonfatal overdoses represent a substantial burden on individuals and health systems and may provide earlier signals of emerging overdose risks (Vivolo-Kantor et al., 2020). Nonfatal overdose is a key indicator of risk for future overdose. Prior studies indicate that 7% of people who use opioids experience recurrent nonfatal overdoses within the same year, and about 1% die from a subsequent overdose within the following year (Karmali et al., 2020; Olfson et al., 2018). Additionally, increases in nonfatal overdose events have been associated with higher concurrent overdose mortality, with each additional nonfatal overdose linked to a 48% higher fatal overdose rate (Skinner et al., 2026). Emergency department (ED) visits for suspected overdoses capture a large share of overdose morbidity and provide opportunity for surveillance and prevention (Vivolo-Kantor et al., 2020). ED visits for overdose are also associated with substantial healthcare costs and resource utilization. Prior studies estimate that overdose-related ED visits and hospitalizations contribute $5 billion annually in medical expenditures in the US, placing significant strain on healthcare systems and public budgets (Langabeer et al., 2021).

At the same time, monitoring changes in the illicit drug supply has become a priority for public health and drug policy (Cano et al., 2024; Dahlen et al., 2026). Data from forensic laboratory analyses of seized substances, such as those compiled in the National Forensic Laboratory Information System (NFLIS), provide one approach to tracking the presence and spread of fentanyl in drug markets (Cano et al., 2024; Park et al., 2021). These data are increasingly used as proxy indicators of changes in the illicit drug supply and may provide timely signals of emerging drug market trends (Cano et al., 2023; Dahlen et al., 2026; Khezri et al., 2026). For example, Dahlen et al. reported that recent declines in opioid overdose mortality were accompanied by declines in the proportion of fentanyl reports in illicit drug seizures (Dahlen et al., 2026), and Vangelov et al. presented evidence suggesting that broader disruptions in fentanyl supply dynamics may have contributed to declining overdose deaths in the US and Canada (Vangelov et al., 2026).

Despite increasing interest in drug supply surveillance, most studies have focused on associations with overdose mortality, and few have examined how variation in fentanyl presence in the illicit drug supply is associated with nonfatal overdose outcomes (Cano et al., 2023; Dahlen et al., 2026; Khezri et al., 2026). Understanding these associations may help identify early warning signals for emerging overdose risks and inform public health preparedness. In this study, we examine the association between the proportion of fentanyl reports in illicit drug seizures and state-level rates of nonfatal overdose ED visits in the US from 2021 through 2024. We further evaluate these associations across multiple drug-specific overdose categories to assess whether fentanyl presence in the drug supply differentially shapes patterns of overdose morbidity across substance classes.

## Methods

### Study design and data sources

We conducted a longitudinal ecological analysis of 40 US states from 2021 through 2024 using state-year units. State-level annual rates of nonfatal overdose ED visits were obtained from the Centers for Disease Control and Prevention’s Drug Overdose Surveillance and Epidemiology Syndromic Surveillance System (DOSE-SYS) (Centers for Disease Control & Prevention, 2026b). At the time of analysis, 47 states and the District of Columbia reported syndromic surveillance data to DOSE-SYS. States with incomplete or missing overdose rate data during the study period were excluded (*n* = 7). After these exclusions, the analytic dataset included 40 states.

Data on illicit drug seizure reports were obtained from NFLIS (National Forensic Laboratory Information System 2026), which captures forensic analyses of substances seized by law enforcement agencies. This study used publicly available data and was deemed exempt by the NYU Institutional Review Board. Reporting follows the Strengthening the Reporting of Observational Studies in Epidemiology guidelines (Von Elm et al., 2007).

### Suspected nonfatal overdose emergency department visits

The outcome was annual state-level rates of suspected nonfatal overdose ED visits per 10,000 ED visits from DOSE-SYS, which identifies cases using predefined syndrome definitions applied to electronic health records. Suspected overdoses were identified using diagnostic codes, including ICD-10-CM, ICD-9-CM, and SNOMED CT. We examined all drug-involved overdoses and 7 categories (opioid-, fentanyl-, heroin-, stimulant-, cocaine-, methamphetamine-, and benzodiazepine-involved). Each outcome was modeled separately.

### Fentanyl reports in illicit drug seizures

The primary exposure was the proportion of fentanyl or fentanyl-related substances among all illicit drug seizure reports in each state-year, used as a proxy for fentanyl presence (hereafter referred to as “fentanyl proportion”). Using NFLIS drug report data, we calculated the annual proportion of seizure reports in each state that contained fentanyl or fentanyl-related compounds relative to the total number of drug seizure reports. Drug reports reflect laboratory-confirmed substances in seized exhibits. Prior studies have used the share of fentanyl reports in seizure data as an indicator of changes in the illicit drug supply and its association with overdose outcomes (Cano et al., 2023; Dahlen et al., 2026; Zoorob et al., 2024).

### Covariates

Covariates were selected a priori based on their potential associations with illicit drug market characteristics and overdose morbidity (Bruzelius et al., 2024; Cano et al., 2024; Dasgupta et al., 2018). State-level sociodemographic covariates were obtained using 1-year American Community Survey estimates from 2021 through 2024. We extracted state-level demographic characteristics including median age, percent female, total population, percent non-Hispanic White, percent non-Hispanic Black, and percent Hispanic. Socioeconomic factors included percent without health insurance coverage, percent living below the federal poverty level, and percent participating in the Supplemental Nutrition Assistance Program (SNAP). We additionally included a state-level measure of pharmacy-based naloxone dispensing rates obtained from the CDC State Naloxone Dispensing Rate dataset, scaled to represent the number of naloxone prescriptions dispensed per 1000 persons (Centers for Disease Control & Prevention, 2026a). Total population was natural log-transformed. Census region was categorized as Northeast, Midwest, South, or West.

### Statistical analysis

The final analytic dataset included 160 state-year observations from 40 states observed annually between 2021 and 2024 for most analyses. Because of minor missing data, sample size varied slightly across outcome-specific models (Table 1). We used generalized estimating equations with a Gaussian distribution and identity link to model state-level nonfatal overdose ED visit rates (Liang & Zeger, 1986). An exchangeable correlation structure accounted for within-state correlation, with robust standard errors clustered by state. For each outcome, we estimated three models representing sequential covariate adjustment. Model 1 included the fentanyl proportion, natural log-transformed state population, calendar year indicators, and US Census region. Model 2 additionally adjusted for racial and ethnic composition. Model 3 further adjusted for socioeconomic indicators, including percent living below the federal poverty level, percent SNAP participation, and percent uninsured. Model 4 additionally adjusted for state-level naloxone dispensing rates (per 1000 persons). To enhance interpretability, the exposure was scaled so coefficients represent the change in ED visit rates per 10–percentage point increase in fentanyl seizure reports. Results are presented as regression coefficients with 95% confidence intervals (CIs). All analyses were conducted in Stata version 18 (StataCorp LLC, College Station, TX). A two-sided *p* < 0.05 was considered statistically significant. We did not adjust for multiple comparisons given the exploratory nature of the study and to avoid increasing Type II error (Rothman, 1990).

## Results

### Descriptive statistics

**Fig. 1.**
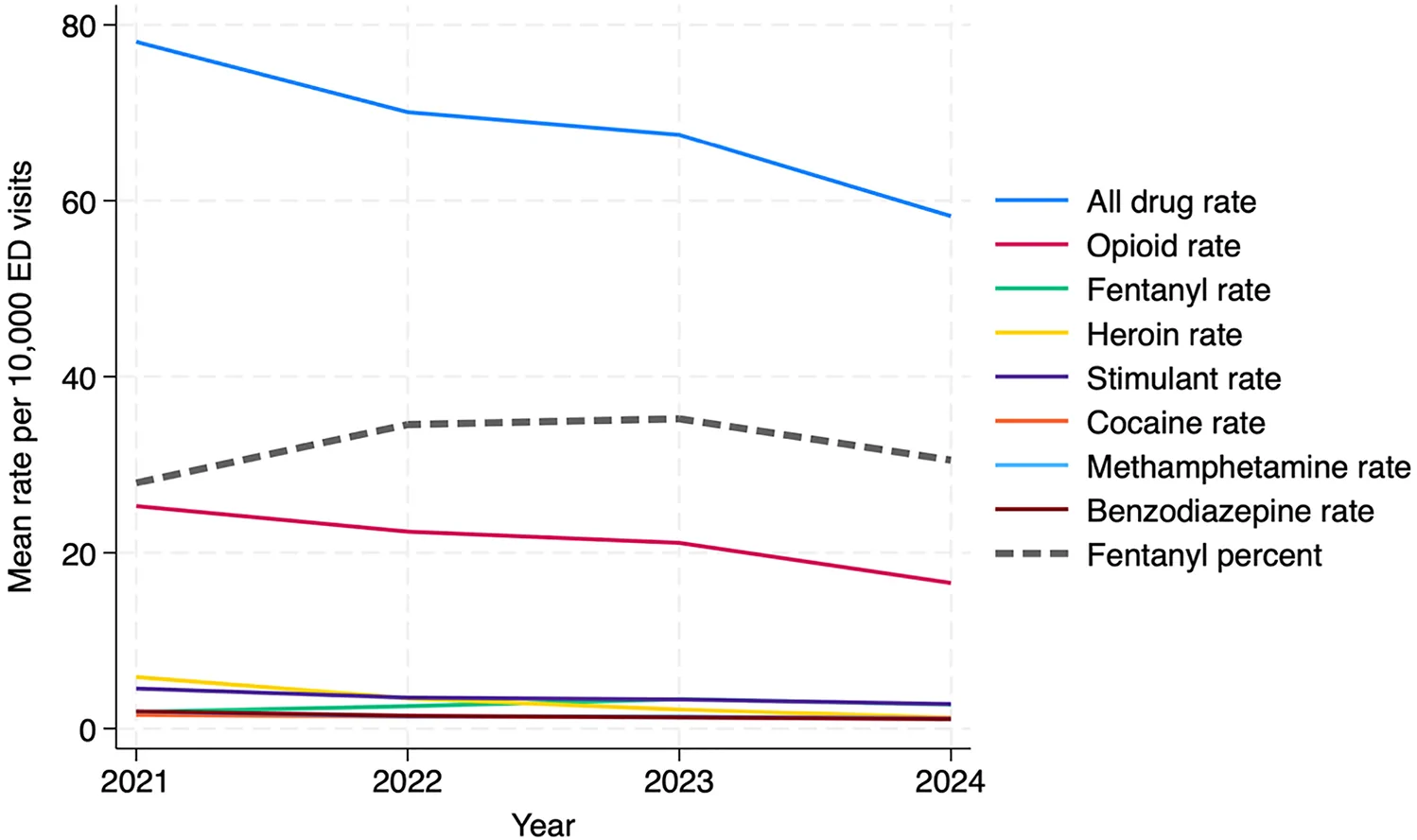
Trends in nonfatal overdose rates and the proportion of fentanyl reports in illicit drug seizures across 40 US states, 2021–2024.

From 2021 to 2024, the mean fentanyl proportion increased from 27.9% in 2021 to 34.6% in 2022 and 35.2% in 2023, before declining to 30.5% in 2024 (Supplementary Table 1). Overall drug-involved ED visit rates declined steadily across the study period, decreasing from 78.1 per 10,000 ED visits in 2021 to 70.1 in 2022, 67.5 in 2023, and 58.3 in 2024. A similar decline was observed for opioid-involved overdose rates, which declined from 25.3 in 2021 to 16.6 in 2024 (Fig. 1).

**Fig. 2.**
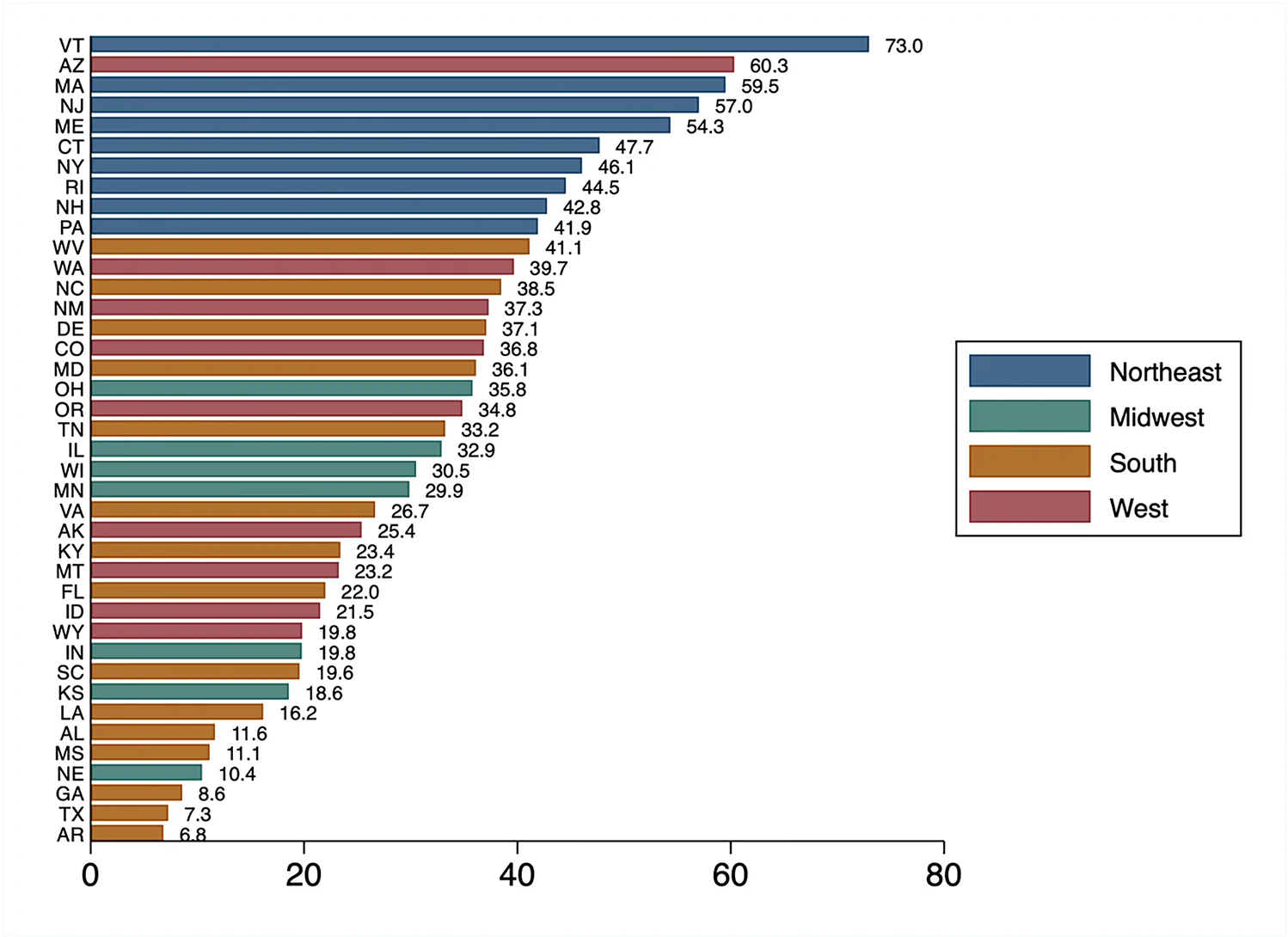
Mean state-level proportion of fentanyl reports in drug seizures across 40 US states, 2021–2024 (overall mean = 32.1%).

The overall mean fentanyl proportion pooled across 2021–2024 was 32.1%, ranging from 6.8% in Arkansas and 7.3% in Texas to 73.0% in Vermont (Fig. 2). Northeastern states showed high fentanyl proportions, including Vermont (73.0%), Massachusetts (59.5%), New Jersey (57.0%), Maine (54.3%). Arizona also showed a high mean proportion (60.3%). Lower proportions were observed in parts of the South and Midwest, including Arkansas (6.8%), Texas (7.3%), Georgia (8.6%), and Nebraska (10.4%). Western and Midwestern states generally demonstrated moderate levels, including Colorado (36.8%), Washington (39.7%), Illinois (32.9%), and Wisconsin (30.5%).

### Fentanyl proportion and nonfatal overdose emergency department visits

Results are shown in Table 1, with full model outputs in Supplementary Tables 2–9. For opioid-involved overdoses, higher fentanyl proportion was consistently associated with higher ED visit rates across all models. In Model 1, a 10 percentage-point increase in fentanyl proportion was significantly associated with a 2.37 increase (95% CI: 0.92, 3.82) in opioid-involved overdose ED visit rates per 10,000 ED visits, adjusting for natural log-transformed state population, calendar year indicators, and US Census region. This association strengthened to a 2.46 increase (95% CI: 1.01, 3.91) when additionally adjusting for demographic factors in Model 2 but then attenuated to a 2.18 increase when accounting for socioeconomic factors in Model 3 (95% CI: 0.90, 3.46). This estimate remained unchanged with the addition of pharmacy-based naloxone dispensing rates in Model 4 (2.18 increase, 95% CI: 0.89, 3.46).

A similar pattern was observed for fentanyl-involved overdose ED visit rates (Table 1). In Model 1, a 10 percentage-point increase in fentanyl proportion was significantly associated with a 0.80 increase (95% CI: 0.36, 1.24) in fentanyl-involved overdose ED visit rates per 10,000 ED visits, with a slight attenuation in Model 2 (0.79 increase, 95% CI: 0.35, 1.23) and further attenuation in Model 3 (0.73 increase, 95% CI: 0.27, 1.19). This association was sustained in Model 4 (0.73 increase, 95% CI: 0.27, 1.18). For context, the estimated 2.18 increase in opioid-involved overdose ED visit rates per 10,000 ED visits was 10.2% higher than the overall mean opioid-involved overdose ED visit rate during the study period (Supplementary Table 10). The estimated increase in fentanyl-involved overdose ED visit rates was 27.3% higher than the overall mean fentanyl-involved overdose ED visit.

Heroin-involved overdoses were significantly associated with fentanyl proportion in Models 1 and 2 but not in Models 3 and 4. A 10 percentage-point increase in fentanyl proportion significantly correlated with a 0.47 increase (95% CI: 0.02, 0.92) in heroin-involved overdose ED visit rates per 10,000 ED visits in Model 1 and a 0.48 increase (95% CI: 0.06, 0.90) in Model 2. However, this association attenuated to non-significance in Model 3 (0.31 increase, 95% CI: −0.08, 0.70) and remained unchanged in Model 4 (0.31 increase, 95% CI: −0.08, 0.69).

Increases in fentanyl proportions were significantly associated with cocaine-involved overdose ED visits across all models. Controlling for differences in population size, time, and census region, Model 1 indicated that a 10 percentage-point increase in fentanyl proportion was significantly associated with a 0.08 increase in cocaine-involved overdose ED visit rates per 10,000 ED visits. This estimate slightly increased in Model 2 (0.09 increase, 95% CI: 0.02, 0.15) but remained stable at a 0.08 increase in Models 3 and 4 (both: 95% CI: 0.01, 0.15).

Associations with methamphetamine-involved overdose ED visits were negative across all models. Model 1 suggested a 0.35 decrease (95% CI: −0.64, −0.06), which was statistically significant. A similar pattern was shown in Model 2 (0.38 decrease, 95% CI: −0.65, −0.11) but attenuated to non-significance in Model 3 (0.24 decrease, 95% CI: −0.53, 0.05) and Model 4 (0.22 decrease, 95% CI: −0.50, 0.06). Across all models, there was no evidence of a statistically significant association of fentanyl proportion with all drug-involved, all stimulant-involved, and benzodiazepine-involved overdose ED visit rates per 10,000 ED visits.

## Discussion

In this longitudinal ecological analysis of 40 states, higher proportions of fentanyl in illicit drug seizures were associated with higher rates of opioid-involved and fentanyl-involved nonfatal overdose ED visits. Higher proportions of fentanyl were also positively associated with cocaine-involved overdoses, whereas no consistent associations were observed for stimulant-involved or benzodiazepine-involved overdoses. The initially observed associations for heroin-involved and methamphetamine-involved overdose ED visit rates did not remain statistically significant after adjustment for socioeconomic indicators, suggesting that these relationships may be partially explained by structural and socioeconomic differences across states. This is consistent with the evolution of the opioid crisis across three waves, in which synthetic opioids have largely replaced heroin as the primary driver of overdose risk in recent years (Ciccarone, 2019). These findings suggest that variation in fentanyl presence within the illicit drug supply is an important correlate of overdose morbidity at the state level.

These findings align with prior research demonstrating that increasing fentanyl presence in the illicit drug supply has contributed to overdose risk. Several studies have shown that the growing saturation of fentanyl and fentanyl analogs in drug markets is strongly associated with overdose mortality, particularly for opioid-involved deaths (Cano et al., 2023, 2024; Ciccarone, 2019; Dahlen et al., 2026; Khezri et al., 2026; Zoorob, 2019; Zoorob et al., 2024). Our results extend this literature by examining nonfatal overdose outcomes captured through syndromic surveillance, which represent a less frequently studied component of the overdose burden. While most research and policy discussions focus on fatal overdoses, nonfatal overdoses impose substantial clinical, economic, and societal costs, including emergency medical care, hospitalizations, and long-term health consequences (Langabeer et al., 2021). Nonfatal overdoses also represent a critical opportunity for intervention and prevention, as individuals who experience a nonfatal overdose are at elevated risk of subsequent fatal overdose (Olfson et al., 2018; Vivolo-Kantor et al., 2020). At the same time, the relationship between fentanyl presence in the drug supply and overdose morbidity may evolve over time as populations adapt to changing drug market conditions, harm reduction interventions expand, and drug use behaviors shift (Dahlen et al., 2026; Vangelov et al., 2026).

The geographic distribution of fentanyl proportions observed in this study reflects previously documented regional patterns in the evolution of the US drug supply. Northeastern states exhibited some of the highest fentanyl proportions, consistent with earlier evidence that fentanyl initially spread through heroin markets in the Northeast before expanding to other regions of the country (Ciccarone, 2019; O’Donnell, 2017). Prior studies have documented how synthetic opioids gradually displaced heroin in many Northeastern markets and subsequently diffused westward and southward through existing drug trafficking networks (O’Donnell, 2017; Pardo, Caulkins et al., 2019, 2019; Zoorob, 2019). More recent work has also shown that the polysubstance profile of fentanyl-involved overdoses varies across regions, with cocaine-fentanyl co-involvement more prominent in the Northeast and methamphetamine-fentanyl co-involvement more common in many Western states, underscoring the broader geographic heterogeneity of fentanyl-related overdose risk (Friedman & Shover, 2023). Although fentanyl is now present across most US drug markets (Friedman & Shover, 2023), the variation observed in this study suggests that regional differences in fentanyl saturation persist. Such geographic variation may reflect differences in drug trafficking routes, regional drug supply chains, and local patterns of drug use.

The positive association between fentanyl proportion and cocaine-involved overdoses is also consistent with emerging evidence that fentanyl contamination and co-use has expanded beyond opioid markets. Increasingly, fentanyl has been detected in stimulant supplies, including cocaine, raising concerns that individuals who do not intentionally use opioids may be exposed to fentanyl unintentionally (Park et al., 2021; Spencer et al., 2023). National evidence further suggests that stimulant-fentanyl co-involvement has become a prominent feature of the evolving overdose crisis, particularly in states where cocaine-fentanyl combinations predominate (Friedman & Shover, 2023). Although the magnitude of the association observed in this study was smaller than that observed for opioid-involved overdoses, these findings highlight fentanyl’s influence across drug categories.

However, the associations for heroin-involved and methamphetamine-involved overdose ED visit rates were attenuated after adjustment for socioeconomic indicators. This pattern suggests that the relationships between fentanyl market saturation and these overdose outcomes may be explained, at least in part, by broader structural and socioeconomic conditions (Dasgupta et al., 2018; Friedman & Hansen, 2024). It may also reflect regional variation in how fentanyl is incorporated into local drug markets, including differences in heroin–fentanyl versus methamphetamine–fentanyl combinations across states (Friedman & Shover, 2023). Prior qualitative research found that some people who co-use opioids and methamphetamine perceive methamphetamine as helping prevent overdose or manage opioid withdrawal (Ondocsin et al., 2023), while Rosenblum et al. also reported that greater methamphetamine presence in Ohio crime lab data was not positively associated with overdose mortality and was negatively associated with mortality in some settings (Rosenblum et al., 2024). These findings underscore the importance of interpreting drug market indicators within their broader social and structural context.

This study also has several implications for drug market surveillance and overdose prevention efforts. Monitoring changes in the illicit drug supply may provide early warning signals for shifts in overdose risk (Pardo, Caulkins et al., 2019). Surveillance systems for detecting changes in the drug supply are essential for anticipating and preventing overdose risk. While seizure data provide one indicator of drug market composition, they may reflect reactive enforcement practices rather than real-time changes in drug availability. Expanding surveillance approaches, including drug checking services, point-of-use testing, and sentinel surveillance systems implemented by public health agencies, may allow earlier detection of emerging drug threats (Ciccarone, 2021; Pardo, Caulkins et al., 2019). Diversifying these surveillance strategies could improve the timeliness and effectiveness of public health responses, including targeted naloxone distribution, harm reduction outreach, disseminating warnings about contaminated drug supplies, and preparing emergency departments and health systems for potential increases in overdose presentations (Bennett & Elliott, 2021; Carroll et al., 2018). Integrating drug supply surveillance with overdose surveillance systems may therefore strengthen public health preparedness and response to emerging drug market trends (Cano et al., 2024; Wolff et al., 2022).

This study has several limitations. First, the analysis was conducted at the state level and is therefore subject to the ecological fallacy. The observed associations reflect aggregate state-level patterns and do not imply that the individuals exposed to fentanyl-containing drug supplies are the same individuals who experience overdose events. State-level associations may also be influenced by unmeasured differences in healthcare access, drug market dynamics, law enforcement practices, and harm reduction infrastructure across states. Second, outcomes were based on suspected nonfatal overdose ED visits from syndromic surveillance using preliminary codes and may not reflect final diagnoses. Limited toxicological testing may lead to underreporting or misclassification of drugs involved. Drug categories are not mutually exclusive, and some overdoses may involve multiple substances. These data may also underestimate events due to incomplete documentation or coding errors. Third, many nonfatal overdoses may not result in ED visits due to community-based overdose responses and naloxone administration outside healthcare settings (Saloner et al., 2025). Therefore, the outcome likely represents an underestimation of the entire nonfatal overdose burden and should be interpreted as a measure of overdoses that result in ED utilization. Although we adjusted for pharmacy-based naloxone dispensing rates, this measure likely underestimates broader naloxone availability because community-based distribution and overdose response efforts are not fully captured. Finally, NFLIS seizure data reflect substances analyzed after law enforcement seizures and may not represent the broader drug supply, as they are influenced by enforcement, reporting, and testing practices. Additionally, consistent with prior research (Dahlen et al., 2026), the exposure measure did not capture fentanyl purity or other characteristics of the drug supply that may also influence overdose risk.

Despite these limitations, this study provides new evidence linking changes in the illicit drug supply to nonfatal overdose morbidity across 40 states. By integrating seizure and ED surveillance data, it contributes to understanding how drug market shifts influence overdose-related healthcare use. Future research should use additional surveillance data and finer geographic levels to better understand how drug supply changes affect overdose risk. Additional work is needed to better characterize the proportion of nonfatal overdoses managed by emergency services versus in the community, how this varies across settings, and how to incorporate more comprehensive measures of naloxone distribution and other harm reduction interventions.

## Conclusions

This study found that higher proportions of fentanyl reports in illicit drug seizures were associated with higher rates of opioid-involved and fentanyl-involved nonfatal overdose ED visits. The findings suggest that changes in the composition of the illicit drug supply are reflected in patterns of overdose morbidity and health service utilization. Monitoring shifts in drug supply composition may help inform timely public health responses. Integrating diverse drug supply surveillance systems with overdose monitoring systems could improve early detection of emerging drug threats and guide targeted prevention efforts.

## Supplementary Material

Appendix

Supplementary material associated with this article can be found, in the online version, at doi:10.1016/j.drugpo.2026.105382.

## Figures and Tables

**Table 1.** Adjusted associations between the proportion of fentanyl reports in illicit drug seizures and nonfatal overdose emergency department (ED) visits, 2021–2024.

Panel A. Associations with all drugs and opioid-related overdose ED visits								
	*All Drugs*		*Opioid-involved*		*Fentanyl-involved*		*Heroin-involved*	
	b (95% CI)	p value	b (95% CI)	p value	b (95% CI)	p value	b (95% CI)	p value
*Model 1*	1.56 (−0.42, 3.53)	0.12	**2.37 (0.92, 3.82)**	**0.001**	**0.80 (0.36, 1.24)**	**<0.001**	**0.47 (0.02, 0.92)**	**0.043**
*Model 2*	1.74 (−0.26, 3.73)	0.088	**2.46 (1.01, 3.91)**	**0.001**	**0.79 (0.35, 1.23)**	**<0.001**	**0.48 (0.06, 0.90)**	**0.025**
*Model 3*	1.44 (−0.31, 3.18)	0.11	**2.18 (0.90, 3.46)**	**0.001**	**0.73 (0.27, 1.19)**	**0.002**	0.31 (−0.08, 0.70)	0.12
*Model 4*	1.45 (−0.30, 3.19)	0.10	**2.18 (0.89, 3.46)**	**0.001**	**0.73 (0.27, 1.18)**	**0.002**	0.31 (−0.08, 0.69)	0.12
Panel B. Associations with stimulant and benzodiazepine overdose ED visits								
	*All Stimulants*		*Cocaine-involved*		*Methamphetamine-involved*		*Benzodiazepine-involved*	
	b (95% CI)	p value	b (95% CI)	p value	b (95% CI)	p value	b (95% CI)	p value
*Model 1*	−0.25 (−0.54, 0.04)	0.095	**0.08 (0.01, 0.15)**	**0.024**	**−0.35 (−0.64, -0.06)**	**0.019**	−0.02 (−0.08, 0.04)	0.47
*Model 2*	−0.25 (−0.53, 0.03)	0.077	**0.09 (0.02, 0.15)**	**0.008**	**−0.38 (−0.65, -0.11)**	**0.006**	−0.03 (−0.09, 0.03)	0.39
*Model 3*	−0.20 (−0.50, 0.10)	0.19	**0.08 (0.01, 0.15)**	**0.025**	−0.24 (−0.53, 0.05)	0.11	−0.03 (−0.10, 0.03)	0.29
*Model 4*	−0.21 (−0.51, 0.10)	0.18	**0.08 (0.01, 0.15)**	**0.024**	−0.22 (−0.50, 0.06)	0.12	−0.03 (−0.10, 0.03)	0.30

Regression coefficients represent the expected change in nonfatal overdose ED visits per 10,000 ED visits associated with a 10 percentage-point increase in the proportion of fentanyl seizure reports. Bold font indicates *p*<0.05

**Model 1:** log-transformed population size, year, census region

**Model 2:** Model 1 + race/ethnicity composition

**Model 3:** Model 2 + socioeconomic indicators

**Model 4:** Model 3 + naloxone dispensing rate

Sample size (state-years; states): All drugs 160;40; opioid 160;40; fentanyl 155;39; heroin 143;39; stimulants 160;40; cocaine 149;39; methamphetamine 145;37; benzodiazepine 158;40

## Data Availability

All data used in this study are publicly available. Nonfatal overdose emergency department visit data were obtained from the Centers for Disease Control and Prevention (CDC) Drug Overdose Surveillance and Epidemiology (DOSE) system. Data on fentanyl seizure reports were obtained from the National Forensic Laboratory Information System (NFLIS). State-level demographic and socioeconomic covariates were derived from the American Community Survey (ACS) 1-year estimates. State naloxone dispensing rates were obtained from the CDC Overdose Prevention Naloxone Dispensing Rate dataset. Links to these publicly available sources are provided in the references.
