# Supplementary material for: Proportion of fentanyl reports in illicit drug seizures and nonfatal overdose emergency department visits in the United States, 2021–2024
Source: Int J Drug Policy. Author manuscript; Available in PMC 2026 Jul 23. (PMC13395472; doi:10.1016/j.drugpo.2026.105382)
Supplement: Appendix [file NIHMS2188641-supplement-Appendix.docx]

**Supplementary Table 1.** Proportion of fentanyl reports in drug seizures (%) by year, 2021–2024.

| **Year** | **Mean** | **Median** | **25th percentile** | **75th percentile** | **Minimum value** | **Maximum value** |
| --- | --- | --- | --- | --- | --- | --- |
| 2021 | 27.9 | 23.7 | 11.7 | 40.0 | 3.8 | 84.1 |
| 2022 | 34.6 | 35.1 | 21.5 | 43.3 | 6.3 | 84.5 |
| 2023 | 35.2 | 34.6 | 22.8 | 44.4 | 8.2 | 82.4 |
| 2024 | 30.5 | 33.0 | 20.3 | 40.5 | 5.8 | 58.3 |
| Total | 32.1 | 32.3 | 20.1 | 42.4 | 3.8 | 84.5 |

**Supplementary Table 2.** Adjusted associations between the proportion of fentanyl reports in illicit drug seizures and all drug-involved nonfatal overdose emergency department (ED) visits, 2021-2024. Regression coefficients represent the expected change in all drug-involved nonfatal overdose ED visits per 10,000 ED visits associated with a 10 percentage-point increase in the proportion of fentanyl seizure reports.

|  | **Model 1** | | **Model 2** | | **Model 3** | | **Model 4** | |
| --- | --- | --- | --- | --- | --- | --- | --- | --- |
|  | b (95% CI) | p value | b (95% CI) | p value | b (95% CI) | p value | b (95% CI) | p value |
| **Fentanyl proportion** | 1.56 (-0.42, 3.53) | 0.122 | 1.74 (-0.26, 3.73) | 0.088 | 1.44 (-0.31, 3.18) | 0.107 | 1.45 (-0.30, 3.19) | 0.104 |
| **Log(total population)** | 1.87 (-2.19, 5.93) | 0.367 | 3.26 (-1.00, 7.53) | 0.133 | 2.93 (-0.63, 6.49) | 0.107 | 2.90 (-0.67, 6.47) | 0.111 |
| **Year** |  |  |  |  |  |  |  |  |
| 2021 (reference) |  |  |  |  |  |  |  |  |
| 2022 | -9.02 (-11.28, -6.76) | <0.001 | -8.91 (-11.22, -6.61) | <0.001 | -9.46 (-11.95, -6.97) | <0.001 | -9.39 (-11.93, -6.85) | <0.001 |
| 2023 | -11.71 (-14.38, -9.05) | <0.001 | -11.70 (-14.37, -9.02) | <0.001 | -12.31 (-15.18, -9.45) | <0.001 | -12.15 (-15.12, -9.18) | <0.001 |
| 2024 | -20.24 (-23.45, -17.03) | <0.001 | -20.50 (-23.73, -17.26) | <0.001 | -20.75 (-24.06, -17.45) | <0.001 | -20.73 (-24.02, -17.43) | <0.001 |
| **Region** |  |  |  |  |  |  |  |  |
| Northeast (reference) |  |  |  |  |  |  |  |  |
| Midwest | 0.60 (-12.46, 13.65) | 0.93 | 2.48 (-9.70, 14.67) | 0.690 | 5.51 (-7.47, 18.48) | 0.406 | 5.48 (-7.53, 18.48) | 0.409 |
| South | 2.57 (-9.26, 14.40) | 0.67 | 8.03 (-3.79, 19.85) | 0.183 | 13.93 (-1.28, 29.14) | 0.073 | 14.08 (-0.77, 28.93) | 0.063 |
| West | 11.04 (-0.09, 22.16) | 0.052 | 9.42 (1.04, 17.80) | 0.028 | 14.85 (6.40, 23.31) | 0.001 | 14.91 (6.62, 23.20) | <0.001 |
| **Percent White** |  |  | -1.43 (-2.24, -0.62) | 0.001 | -1.34 (-1.90, -0.79) | <0.001 | -1.35 (-1.90, -0.79) | <0.001 |
| **Percent Black** |  |  | -1.64 (-2.72, -0.57) | 0.003 | -1.51 (-2.33, -0.69) | <0.001 | -1.52 (-2.34, -0.70) | <0.001 |
| **Percent Hispanic** |  |  | -1.53 (-2.24, -0.81) | <0.001 | -1.30 (-1.82, -0.78) | <0.001 | -1.30 (-1.82, -0.77) | <0.001 |
| **Percent living below the federal poverty level** |  |  |  |  | -0.36 (-2.18, 1.46) | 0.70 | -0.32 (-2.24, 1.61) | 0.745 |
| **Percent participating in the Supplemental Nutrition Assistance Program** |  |  |  |  | 0.47 (-0.76, 1.69) | 0.46 | 0.45 (-0.83, 1.73) | 0.494 |
| **Percent without health insurance coverage** |  |  |  |  | -1.54 (-3.39, 0.30) | 0.10 | -1.57 (-3.40, 0.26) | 0.092 |
| **Naloxone dispensing rate (per 1,000 persons)** |  |  |  |  |  |  | -0.06 (-0.33, 0.22) | 0.673 |

**Supplementary Table 3.** Adjusted associations between the proportion of fentanyl reports in illicit drug seizures and opioid-involved nonfatal overdose emergency department (ED) visits, 2021-2024. Regression coefficients represent the expected change in opioid-involved nonfatal overdose ED visits per 10,000 ED visits associated with a 10 percentage-point increase in the proportion of fentanyl seizure reports.

|  | **Model 1** | | **Model 2** | | **Model 3** | | **Model 4** | |
| --- | --- | --- | --- | --- | --- | --- | --- | --- |
|  | b (95% CI) | p value | b (95% CI) | p value | b (95% CI) | p value | b (95% CI) | p value |
| **Fentanyl proportion** | 2.37 (0.92, 3.82) | 0.001 | 2.46 (1.01, 3.91) | 0.001 | 2.18 (0.90, 3.46) | 0.001 | 2.18 (0.89, 3.46) | 0.001 |
| **Log(total population)** | 0.66 (-2.23, 3.55) | 0.656 | 0.93 (-2.29, 4.15) | 0.572 | 0.42 (-2.14, 2.97) | 0.749 | 0.43 (-2.14, 3.01) | 0.742 |
| **Year** |  |  |  |  |  |  |  |  |
| 2021 (reference) |  |  |  |  |  |  |  |  |
| 2022 | -4.48 (-6.02, -2.94) | <0.001 | -4.43 (-5.99, -2.87) | <0.001 | -4.83 (-6.38, -3.27) | <0.001 | -4.86 (-6.43, -3.29) | <0.001 |
| 2023 | -5.91 (-7.99, -3.83) | <0.001 | -5.94 (-8.04, -3.85) | <0.001 | -6.30 (-8.39, -4.21) | <0.001 | -6.38 (-8.46, -4.30) | <0.001 |
| 2024 | -9.35 (-11.54, -7.17) | <0.001 | -9.57 (-11.80, -7.35) | <0.001 | -9.60 (-11.75, -7.45) | <0.001 | -9.61 (-11.74, -7.48) | <0.001 |
| **Region** |  |  |  |  |  |  |  |  |
| Northeast (reference) |  |  |  |  |  |  |  |  |
| Midwest | 1.62 (-6.18, 9.42) | 0.684 | 3.01 (-4.71, 10.72) | 0.445 | 6.49 (-1.35, 14.33) | 0.105 | 6.50 (-1.36, 14.37) | 0.105 |
| South | 5.14 (-1.57, 11.85) | 0.133 | 7.90 (0.06, 15.74) | 0.048 | 13.48 (4.61, 22.34) | 0.003 | 13.40 (4.66, 22.14) | 0.003 |
| West | 5.49 (-1.81, 12.79) | 0.141 | 4.06 (-2.43, 10.56) | 0.220 | 9.16 (4.69, 13.64) | <0.001 | 9.13 (4.71, 13.55) | <0.001 |
| **Percent White** |  |  | -0.80 (-1.33, -0.27) | 0.003 | -0.69 (-1.03, -0.34) | <0.001 | -0.68 (-1.03, -0.34) | <0.001 |
| **Percent Black** |  |  | -0.88 (-1.54, -0.21) | 0.010 | -0.73 (-1.20, -0.25) | 0.003 | -0.72 (-1.20, -0.25) | 0.003 |
| **Percent Hispanic** |  |  | -0.78 (-1.27, -0.29) | 0.002 | -0.57 (-0.92, -0.22) | 0.002 | -0.57 (-0.92, -0.22) | 0.002 |
| **Percent living below the federal poverty level** |  |  |  |  | -0.63 (-1.55, 0.28) | 0.175 | -0.65 (-1.66, 0.35) | 0.203 |
| **Percent participating in the Supplemental Nutrition Assistance Program** |  |  |  |  | 0.82 (0.15, 1.50) | 0.017 | 0.83 (0.12, 1.55) | 0.022 |
| **Percent without health insurance coverage** |  |  |  |  | -1.20 (-2.24, -0.16) | 0.024 | -1.19 (-2.23, -0.14) | 0.026 |
| **Naloxone dispensing rate (per 1,000 persons)** |  |  |  |  |  |  | 0.03 (-0.21, 0.27) | 0.807 |

**Supplementary Table 4.** Adjusted associations between the proportion of fentanyl reports in illicit drug seizures and fentanyl-involved nonfatal overdose emergency department (ED) visits, 2021-2024. Regression coefficients represent the expected change in fentanyl-involved nonfatal overdose ED visits per 10,000 ED visits associated with a 10 percentage-point increase in the proportion of fentanyl seizure reports.

|  | **Model 1** | | **Model 2** | | **Model 3** | | **Model 4** | |
| --- | --- | --- | --- | --- | --- | --- | --- | --- |
|  | b (95% CI) | p value | b (95% CI) | p value | b (95% CI) | p value | b (95% CI) | p value |
| **Fentanyl proportion** | 0.80 (0.36, 1.24) | <0.001 | 0.79 (0.35, 1.23) | <0.001 | 0.73 (0.27, 1.19) | 0.002 | 0.73 (0.27, 1.18) | 0.002 |
| **Log(total population)** | 0.09 (-0.44, 0.63) | 0.739 | 0.06 (-0.63, 0.74) | 0.874 | 0.02 (-0.69, 0.73) | 0.954 | 0.03 (-0.68, 0.73) | 0.942 |
| **Year** |  |  |  |  |  |  |  |  |
| 2021 (reference) |  |  |  |  |  |  |  |  |
| 2022 | 0.15 (-0.18, 0.49) | 0.367 | 0.17 (-0.18, 0.51) | 0.350 | 0.18 (-0.18, 0.54) | 0.329 | 0.17 (-0.21, 0.54) | 0.386 |
| 2023 | 0.88 (0.22, 1.55) | 0.009 | 0.88 (0.20, 1.57) | 0.012 | 0.91 (0.21, 1.61) | 0.011 | 0.88 (0.15, 1.62) | 0.019 |
| 2024 | 0.66 (-0.06, 1.37) | 0.073 | 0.63 (-0.11, 1.37) | 0.095 | 0.66 (-0.11, 1.43) | 0.095 | 0.65 (-0.12, 1.43) | 0.099 |
| **Region** |  |  |  |  |  |  |  |  |
| Northeast (reference) |  |  |  |  |  |  |  |  |
| Midwest | 1.52 (0.01, 3.02) | 0.048 | 1.66 (0.25, 3.07) | 0.021 | 1.85 (0.40, 3.31) | 0.013 | 1.86 (0.41, 3.31) | 0.012 |
| South | 2.78 (1.30, 4.25) | <0.001 | 2.99 (1.46, 4.51) | <0.001 | 3.21 (1.56, 4.85) | <0.001 | 3.18 (1.50, 4.86) | <0.001 |
| West | 4.72 (2.74, 6.69) | <0.001 | 4.47 (2.39, 6.55) | <0.001 | 4.72 (2.86, 6.57) | <0.001 | 4.71 (2.84, 6.57) | <0.001 |
| **Percent White** |  |  | -0.07 (-0.18, 0.04) | 0.235 | -0.06 (-0.17, 0.05) | 0.280 | -0.06 (-0.17, 0.05) | 0.282 |
| **Percent Black** |  |  | -0.07 (-0.21, 0.06) | 0.277 | -0.07 (-0.20, 0.07) | 0.340 | -0.06 (-0.20, 0.07) | 0.349 |
| **Percent Hispanic** |  |  | -0.05 (-0.17, 0.06) | 0.357 | -0.04 (-0.16, 0.07) | 0.460 | -0.05 (-0.16, 0.07) | 0.450 |
| **Percent living below the federal poverty level** |  |  |  |  | -0.05 (-0.32, 0.21) | 0.699 | -0.06 (-0.37, 0.24) | 0.686 |
| **Percent participating in the Supplemental Nutrition Assistance Program** |  |  |  |  | 0.08 (-0.11, 0.27) | 0.408 | 0.09 (-0.12, 0.29) | 0.413 |
| **Percent without health insurance coverage** |  |  |  |  | -0.06 (-0.27, 0.15) | 0.563 | -0.05 (-0.27, 0.16) | 0.617 |
| **Naloxone dispensing rate (per 1,000 persons)** |  |  |  |  |  |  | 0.01 (-0.06, 0.09) | 0.774 |

**Supplementary Table 5.** Adjusted associations between the proportion of fentanyl reports in illicit drug seizures and heroin-involved nonfatal overdose emergency department (ED) visits, 2021-2024. Regression coefficients represent the expected change in heroin-involved nonfatal overdose ED visits per 10,000 ED visits associated with a 10 percentage-point increase in the proportion of fentanyl seizure reports.

|  | **Model 1** | | **Model 2** | | **Model 3** | | **Model 4** | |
| --- | --- | --- | --- | --- | --- | --- | --- | --- |
|  | b (95% CI) | p value | b (95% CI) | p value | b (95% CI) | p value | b (95% CI) | p value |
| **Fentanyl proportion** | 0.47 (0.02, 0.92) | 0.043 | 0.48 (0.06, 0.90) | 0.025 | 0.31 (-0.08, 0.70) | 0.115 | 0.31 (-0.08, 0.69) | 0.122 |
| **Log(total population)** | -0.25 (-0.93, 0.42) | 0.459 | 0.03 (-0.85, 0.91) | 0.952 | -0.00 (-0.82, 0.81) | 0.99 | -0.03 (-0.82, 0.77) | 0.946 |
| **Year** |  |  |  |  |  |  |  |  |
| 2021 (reference) |  |  |  |  |  |  |  |  |
| 2022 | -2.82 (-3.41, -2.22) | <0.001 | -2.80 (-3.39, -2.21) | <0.001 | -2.83 (-3.41, -2.25) | <0.001 | -2.72 (-3.34, -2.10) | <0.001 |
| 2023 | -4.43 (-5.30, -3.57) | <0.001 | -4.42 (-5.30, -3.54) | <0.001 | -4.46 (-5.35, -3.58) | <0.001 | -4.28 (-5.24, -3.32) | <0.001 |
| 2024 | -5.31 (-6.34, -4.28) | <0.001 | -5.32 (-6.38, -4.26) | <0.001 | -5.34 (-6.42, -4.25) | <0.001 | -5.30 (-6.38, -4.22) | <0.001 |
| **Region** |  |  |  |  |  |  |  |  |
| Northeast (reference) |  |  |  |  |  |  |  |  |
| Midwest | -0.55 (-2.61, 1.50) | 0.598 | -0.50 (-2.52, 1.52) | 0.629 | -0.09 (-2.06, 1.88) | 0.930 | -0.16 (-2.18, 1.85) | 0.874 |
| South | 1.60 (-0.39, 3.60) | 0.115 | 2.27 (0.14, 4.41) | 0.036 | 3.01 (0.57, 5.44) | 0.015 | 3.15 (0.54, 5.77) | 0.018 |
| West | -1.79 (-3.14, -0.44) | 0.009 | -1.88 (-3.53, -0.23) | 0.025 | -1.21 (-2.91, 0.49) | 0.163 | -1.13 (-2.86, 0.60) | 0.200 |
| **Percent White** |  |  | -0.15 (-0.22, -0.07) | <0.001 | -0.14 (-0.25, -0.03) | 0.011 | -0.15 (-0.25, -0.04) | 0.007 |
| **Percent Black** |  |  | -0.18 (-0.32, -0.05) | 0.007 | -0.18 (-0.34, -0.02) | 0.024 | -0.19 (-0.34, -0.04) | 0.015 |
| **Percent Hispanic** |  |  | -0.18 (-0.26, -0.10) | <0.001 | -0.15 (-0.24, -0.05) | 0.003 | -0.14 (-0.24, -0.05) | 0.002 |
| **Percent living below the federal poverty level** |  |  |  |  | -0.03 (-0.41, 0.35) | 0.871 | 0.03 (-0.40, 0.45) | 0.900 |
| **Percent participating in the Supplemental Nutrition Assistance Program** |  |  |  |  | 0.12 (-0.16, 0.40) | 0.403 | 0.10 (-0.19, 0.39) | 0.510 |
| **Percent without health insurance coverage** |  |  |  |  | -0.29 (-0.71, 0.13) | 0.179 | -0.33 (-0.80, 0.13) | 0.159 |
| **Naloxone dispensing rate (per 1,000 persons)** |  |  |  |  |  |  | -0.08 (-0.26, 0.11) | 0.417 |

**Supplementary Table 6.** Adjusted associations between the proportion of fentanyl reports in illicit drug seizures and stimulant-involved nonfatal overdose emergency department (ED) visits, 2021-2024. Regression coefficients represent the expected change in stimulant-involved nonfatal overdose ED visits per 10,000 ED visits associated with a 10 percentage-point increase in the proportion of fentanyl seizure reports.

|  | **Model 1** | | **Model 2** | | **Model 3** | | **Model 4** | |
| --- | --- | --- | --- | --- | --- | --- | --- | --- |
|  | b (95% CI) | p value | b (95% CI) | p value | b (95% CI) | p value | b (95% CI) | p value |
| **Fentanyl proportion** | -0.25 (-0.54, 0.04) | 0.095 | -0.25 (-0.53, 0.03) | 0.077 | -0.20 (-0.50, 0.10) | 0.191 | -0.21 (-0.51, 0.10) | 0.184 |
| **Log(total population)** | -0.23 (-0.58, 0.12) | 0.203 | -0.21 (-0.55, 0.14) | 0.242 | -0.19 (-0.54, 0.17) | 0.305 | -0.18 (-0.55, 0.19) | 0.346 |
| **Year** |  |  |  |  |  |  |  |  |
| 2021 (reference) |  |  |  |  |  |  |  |  |
| 2022 | -0.87 (-1.21, -0.54) | <0.001 | -0.85 (-1.20, -0.50) | <0.001 | -0.82 (-1.19, -0.46) | <0.001 | -0.84 (-1.22, -0.46) | <0.001 |
| 2023 | -1.06 (-1.48, -0.64) | <0.001 | -1.07 (-1.50, -0.63) | <0.001 | -1.02 (-1.48, -0.56) | <0.001 | -1.06 (-1.58, -0.54) | <0.001 |
| 2024 | -1.70 (-2.21, -1.18) | <0.001 | -1.75 (-2.28, -1.22) | <0.001 | -1.67 (-2.24, -1.11) | <0.001 | -1.68 (-2.25, -1.11) | <0.001 |
| **Region** |  |  |  |  |  |  |  |  |
| Northeast (reference) |  |  |  |  |  |  |  |  |
| Midwest | -0.29 (-1.51, 0.92) | 0.636 | -0.02 (-1.23, 1.18) | 0.968 | -0.08 (-1.33, 1.17) | 0.900 | -0.08 (-1.33, 1.18) | 0.906 |
| South | 0.39 (-0.75, 1.53) | 0.501 | 1.48 (0.24, 2.72) | 0.020 | 1.06 (-0.30, 2.42) | 0.126 | 1.02 (-0.32, 2.36) | 0.136 |
| West | 1.69 (0.53, 2.85) | 0.004 | 1.03 (-0.28, 2.34) | 0.123 | 0.77 (-0.55, 2.09) | 0.251 | 0.75 (-0.59, 2.09) | 0.272 |
| **Percent White** |  |  | -0.12 (-0.19, -0.06) | <0.001 | -0.14 (-0.20, -0.07) | <0.001 | -0.14 (-0.20, -0.07) | <0.001 |
| **Percent Black** |  |  | -0.19 (-0.27, -0.10) | <0.001 | -0.21 (-0.30, -0.12) | <0.001 | -0.21 (-0.29, -0.12) | <0.001 |
| **Percent Hispanic** |  |  | -0.10 (-0.16, -0.04) | 0.001 | -0.12 (-0.18, -0.07) | <0.001 | -0.12 (-0.18, -0.07) | <0.001 |
| **Percent living below the federal poverty level** |  |  |  |  | 0.12 (-0.11, 0.34) | 0.320 | 0.10 (-0.11, 0.32) | 0.342 |
| **Percent participating in the Supplemental Nutrition Assistance Program** |  |  |  |  | -0.01 (-0.17, 0.16) | 0.945 | 0.00 (-0.16, 0.16) | 0.999 |
| **Percent without health insurance coverage** |  |  |  |  | 0.07 (-0.11, 0.26) | 0.429 | 0.08 (-0.10, 0.27) | 0.384 |
| **Naloxone dispensing rate (per 1,000 persons)** |  |  |  |  |  |  | 0.02 (-0.04, 0.08) | 0.585 |

**Supplementary Table 7.** Adjusted associations between the proportion of fentanyl reports in illicit drug seizures and cocaine-involved nonfatal overdose emergency department (ED) visits, 2021-2024. Regression coefficients represent the expected change in cocaine-involved nonfatal overdose ED visits per 10,000 ED visits associated with a 10 percentage-point increase in the proportion of fentanyl seizure reports.

|  | **Model 1** | | **Model 2** | | **Model 3** | | **Model 4** | |
| --- | --- | --- | --- | --- | --- | --- | --- | --- |
|  | b (95% CI) | p value | b (95% CI) | p value | b (95% CI) | p value | b (95% CI) | p value |
| **Fentanyl proportion** | 0.08 (0.01, 0.15) | 0.024 | 0.09 (0.02, 0.15) | 0.008 | 0.08 (0.01, 0.15) | 0.025 | 0.08 (0.01, 0.15) | 0.024 |
| **Log(total population)** | 0.11 (-0.13, 0.34) | 0.366 | 0.00 (-0.22, 0.22) | 0.992 | -0.02 (-0.22, 0.18) | 0.879 | -0.02 (-0.22, 0.19) | 0.877 |
| **Year** |  |  |  |  |  |  |  |  |
| 2021 (reference) |  |  |  |  |  |  |  |  |
| 2022 | -0.26 (-0.35, -0.18) | <0.001 | -0.27 (-0.36, -0.19) | <0.001 | -0.28 (-0.37, -0.19) | <0.001 | -0.28 (-0.38, -0.18) | <0.001 |
| 2023 | -0.25 (-0.42, -0.07) | 0.007 | -0.27 (-0.45, -0.09) | 0.004 | -0.27 (-0.46, -0.07) | 0.007 | -0.26 (-0.47, -0.06) | 0.013 |
| 2024 | -0.37 (-0.55, -0.19) | <0.001 | -0.41 (-0.59, -0.22) | <0.001 | -0.40 (-0.59, -0.21) | <0.001 | -0.40 (-0.59, -0.21) | <0.001 |
| **Region** |  |  |  |  |  |  |  |  |
| Northeast (reference) |  |  |  |  |  |  |  |  |
| Midwest | -0.76 (-1.34, -0.18) | 0.010 | -0.60 (-1.15, -0.05) | 0.033 | -0.45 (-1.02, 0.13) | 0.127 | -0.45 (-1.03, 0.13) | 0.128 |
| South | -0.14 (-0.76, 0.48) | 0.651 | -0.03 (-0.72, 0.66) | 0.927 | 0.17 (-0.65, 0.98) | 0.688 | 0.17 (-0.63, 0.98) | 0.676 |
| West | -0.75 (-1.28, -0.23) | 0.005 | -1.00 (-1.57, -0.43) | 0.001 | -0.87 (-1.42, -0.32) | 0.002 | -0.87 (-1.42, -0.32) | 0.002 |
| **Percent White** |  |  | -0.04 (-0.07, -0.00) | 0.024 | -0.03 (-0.06, 0.00) | 0.055 | -0.03 (-0.07, 0.00) | 0.063 |
| **Percent Black** |  |  | -0.03 (-0.07, 0.01) | 0.171 | -0.03 (-0.07, 0.02) | 0.246 | -0.03 (-0.07, 0.02) | 0.251 |
| **Percent Hispanic** |  |  | -0.01 (-0.04, 0.01) | 0.319 | -0.01 (-0.03, 0.02) | 0.555 | -0.01 (-0.03, 0.02) | 0.560 |
| **Percent living below the federal poverty level** |  |  |  |  | -0.05 (-0.14, 0.05) | 0.333 | -0.04 (-0.15, 0.06) | 0.388 |
| **Percent participating in the Supplemental Nutrition Assistance Program** |  |  |  |  | 0.05 (-0.03, 0.13) | 0.231 | 0.05 (-0.03, 0.13) | 0.249 |
| **Percent without health insurance coverage** |  |  |  |  | -0.02 (-0.15, 0.10) | 0.718 | -0.02 (-0.16, 0.11) | 0.728 |
| **Naloxone dispensing rate (per 1,000 persons)** |  |  |  |  |  |  | -0.00 (-0.04, 0.04) | 0.934 |

**Supplementary Table 8.** Adjusted associations between the proportion of fentanyl reports in illicit drug seizures and methamphetamine-involved nonfatal overdose emergency department (ED) visits, 2021-2024. Regression coefficients represent the expected change in methamphetamine-involved nonfatal overdose ED visits per 10,000 ED visits associated with a 10 percentage-point increase in the proportion of fentanyl seizure reports.

|  | **Model 1** | | **Model 2** | | **Model 3** | | **Model 4** | |
| --- | --- | --- | --- | --- | --- | --- | --- | --- |
|  | b (95% CI) | p value | b (95% CI) | p value | b (95% CI) | p value | b (95% CI) | p value |
| **Fentanyl proportion** | -0.35 (-0.64, -0.06) | 0.019 | -0.38 (-0.65, -0.11) | 0.006 | -0.24 (-0.53, 0.05) | 0.108 | -0.22 (-0.50, 0.06) | 0.122 |
| **Log(total population)** | -0.43 (-0.73, -0.14) | 0.004 | -0.26 (-0.54, 0.03) | 0.077 | -0.20 (-0.38, -0.02) | 0.034 | -0.20 (-0.39, -0.02) | 0.028 |
| **Year** |  |  |  |  |  |  |  |  |
| 2021 (reference) |  |  |  |  |  |  |  |  |
| 2022 | -0.33 (-0.65, -0.01) | 0.044 | -0.29 (-0.64, 0.06) | 0.107 | -0.31 (-0.69, 0.06) | 0.101 | -0.29 (-0.67, 0.10) | 0.150 |
| 2023 | -0.28 (-0.64, 0.08) | 0.128 | -0.25 (-0.64, 0.14) | 0.216 | -0.29 (-0.71, 0.14) | 0.184 | -0.22 (-0.69, 0.26) | 0.376 |
| 2024 | -0.59 (-1.02, -0.16) | 0.007 | -0.60 (-1.06, -0.14) | 0.010 | -0.61 (-1.11, -0.12) | 0.014 | -0.60 (-1.10, -0.10) | 0.019 |
| **Region** |  |  |  |  |  |  |  |  |
| Northeast (reference) |  |  |  |  |  |  |  |  |
| Midwest | 0.06 (-0.93, 1.04) | 0.910 | 0.08 (-0.83, 1.00) | 0.860 | -0.00 (-0.88, 0.87) | 0.991 | -0.02 (-0.88, 0.84) | 0.964 |
| South | -0.25 (-1.20, 0.69) | 0.602 | 0.51 (-0.26, 1.28) | 0.198 | 0.11 (-0.58, 0.79) | 0.758 | 0.18 (-0.46, 0.82) | 0.586 |
| West | 1.31 (0.47, 2.14) | 0.002 | 0.97 (-0.08, 2.03) | 0.069 | 0.66 (-0.15, 1.46) | 0.111 | 0.69 (-0.13, 1.51) | 0.097 |
| **Percent White** |  |  | -0.08 (-0.13, -0.03) | 0.002 | -0.09 (-0.14, -0.05) | <0.001 | -0.09 (-0.14, -0.05) | <0.001 |
| **Percent Black** |  |  | -0.14 (-0.20, -0.08) | <0.001 | -0.16 (-0.22, -0.09) | <0.001 | -0.16 (-0.22, -0.10) | <0.001 |
| **Percent Hispanic** |  |  | -0.09 (-0.14, -0.04) | 0.001 | -0.11 (-0.15, -0.08) | <0.001 | -0.11 (-0.15, -0.07) | <0.001 |
| **Percent living below the federal poverty level** |  |  |  |  | 0.12 (-0.04, 0.29) | 0.129 | 0.15 (0.01, 0.29) | 0.033 |
| **Percent participating in the Supplemental Nutrition Assistance Program** |  |  |  |  | -0.08 (-0.20, 0.03) | 0.147 | -0.10 (-0.20, 0.01) | 0.069 |
| **Percent without health insurance coverage** |  |  |  |  | 0.12 (-0.01, 0.25) | 0.080 | 0.11 (-0.02, 0.23) | 0.096 |
| **Naloxone dispensing rate (per 1,000 persons)** |  |  |  |  |  |  | -0.03 (-0.07, 0.02) | 0.225 |

**Supplementary Table 9.** Adjusted associations between the proportion of fentanyl reports in illicit drug seizures and benzodiazepine-involved nonfatal overdose emergency department (ED) visits, 2021-2024. Regression coefficients represent the expected change in benzodiazepine-involved nonfatal overdose ED visits per 10,000 ED visits associated with a 10 percentage-point increase in the proportion of fentanyl seizure reports.

|  | **Model 1** | | **Model 2** | | **Model 3** | | **Model 4** | |
| --- | --- | --- | --- | --- | --- | --- | --- | --- |
|  | b (95% CI) | p value | b (95% CI) | p value | b (95% CI) | p value | b (95% CI) | p value |
| **Fentanyl proportion** | -0.02 (-0.08, 0.04) | 0.473 | -0.03 (-0.09, 0.03) | 0.390 | -0.03 (-0.10, 0.03) | 0.291 | -0.03 (-0.10, 0.03) | 0.303 |
| **Log(total population)** | -0.03 (-0.16, 0.10) | 0.633 | -0.02 (-0.17, 0.12) | 0.746 | -0.05 (-0.19, 0.09) | 0.462 | -0.05 (-0.19, 0.08) | 0.439 |
| **Year** |  |  |  |  |  |  |  |  |
| 2021 (reference) |  |  |  |  |  |  |  |  |
| 2022 | -0.50 (-0.62, -0.37) | <0.001 | -0.49 (-0.62, -0.36) | <0.001 | -0.49 (-0.61, -0.36) | <0.001 | -0.48 (-0.61, -0.35) | <0.001 |
| 2023 | -0.67 (-0.82, -0.52) | <0.001 | -0.67 (-0.83, -0.52) | <0.001 | -0.66 (-0.81, -0.52) | <0.001 | -0.65 (-0.81, -0.49) | <0.001 |
| 2024 | -0.87 (-1.02, -0.72) | <0.001 | -0.88 (-1.03, -0.73) | <0.001 | -0.88 (-1.03, -0.74) | <0.001 | -0.88 (-1.02, -0.73) | <0.001 |
| **Region** |  |  |  |  |  |  |  |  |
| Northeast (reference) |  |  |  |  |  |  |  |  |
| Midwest | -0.15 (-0.59, 0.29) | 0.500 | -0.12 (-0.57, 0.33) | 0.609 | -0.03 (-0.51, 0.45) | 0.901 | -0.03 (-0.51, 0.45) | 0.896 |
| South | 0.18 (-0.15, 0.51) | 0.287 | 0.49 (0.11, 0.87) | 0.012 | 0.59 (0.14, 1.05) | 0.010 | 0.61 (0.16, 1.06) | 0.008 |
| West | 0.28 (-0.06, 0.62) | 0.105 | 0.10 (-0.29, 0.49) | 0.605 | 0.17 (-0.27, 0.61) | 0.441 | 0.18 (-0.26, 0.62) | 0.427 |
| **Percent White** |  |  | -0.01 (-0.03, 0.02) | 0.550 | -0.00 (-0.03, 0.03) | 0.948 | -0.00 (-0.03, 0.03) | 0.940 |
| **Percent Black** |  |  | -0.03 (-0.07, 0.00) | 0.079 | -0.02 (-0.06, 0.01) | 0.194 | -0.02 (-0.06, 0.01) | 0.191 |
| **Percent Hispanic** |  |  | -0.00 (-0.03, 0.03) | 0.970 | 0.00 (-0.02, 0.03) | 0.708 | 0.01 (-0.02, 0.03) | 0.688 |
| **Percent living below the federal poverty level** |  |  |  |  | -0.07 (-0.14, 0.00) | 0.064 | -0.06 (-0.14, 0.01) | 0.086 |
| **Percent participating in the Supplemental Nutrition Assistance Program** |  |  |  |  | 0.04 (-0.01, 0.10) | 0.110 | 0.04 (-0.01, 0.10) | 0.136 |
| **Percent without health insurance coverage** |  |  |  |  | 0.01 (-0.06, 0.08) | 0.716 | 0.01 (-0.06, 0.08) | 0.773 |
| **Naloxone dispensing rate (per 1,000 persons)** |  |  |  |  |  |  | -0.01 (-0.02, 0.01) | 0.570 |

# **Supplementary Table 10.** Relative interpretation of Model 4 coefficients compared with mean overdose ED visit rates.

| Outcome | Mean ED visit rate | Model 4 coefficient | Relative to mean (%) |
| --- | --- | --- | --- |
| All drug-involved | 68.47 | 1.45 | 2.1% |
| Opioid-involved | 21.35 | 2.18 | 10.2% |
| Fentanyl-involved | 2.67 | 0.73 | 27.3% |
| Heroin-involved | 3.33 | 0.31 | 9.3% |
| All stimulant-involved | 3.58 | -0.21 | -5.9% |
| Cocaine-involved | 1.38 | 0.08 | 5.8% |
| Methamphetamine-involved | 1.46 | -0.22 | -15.1% |
| Benzodiazepine-involved | 1.45 | -0.03 | -2.1% |

Note: Mean overdose emergency department (ED) visit rates were calculated across all state-year observations. Model 4 coefficients represent the estimated change in overdose ED visit rate per 10 percentage-point increase in fentanyl seizure proportion from final adjusted GEE models including demographic, socioeconomic, regional, and naloxone dispensing covariates. Relative percentages were calculated by dividing the coefficient by the corresponding mean outcome rate.
